# An Integrated Lab-on-Chip for Rapid Identification and Simultaneous Differentiation of Tropical Pathogens

**DOI:** 10.1371/journal.pntd.0003043

**Published:** 2014-07-31

**Authors:** Jeslin J. L. Tan, Monica Capozzoli, Mitsuharu Sato, Wanitda Watthanaworawit, Clare L. Ling, Marjorie Mauduit, Benoît Malleret, Anne-Charlotte Grüner, Rosemary Tan, François H. Nosten, Georges Snounou, Laurent Rénia, Lisa F. P. Ng

**Affiliations:** 1 Singapore Immunology Network (SIgN), Agency for Science, Technology and Research (A*STAR), Biopolis, Singapore; 2 CI Group, Molecular Diagnostic Business Unit, Microfluidics Division, ST Microelectronics, Catania, Italy; 3 Veredus Laboratories Pte Ltd, Singapore Science Park, Singapore; 4 Shoklo Malaria Research Unit, Mahidol-Oxford Tropical Medicine Research Unit, Faculty of Tropical Medicine, Mahidol University, Mae Sot, Thailand; 5 Centre for Tropical Medicine, Nuffield Department of Medicine, University of Oxford, Oxford, United Kingdom; 6 Université Pierre et Marie Curie (Paris VI), Centre Hospitalo-Universitaire Pitié-Salpêtrière, Paris, France; 7 INSERM UMR S 945, Paris, France; 8 Department of Biochemistry, Yong Loo Lin School of Medicine, National University of Singapore, Singapore; U.S. Naval Medical Research Unit No. 2, Indonesia

## Abstract

Tropical pathogens often cause febrile illnesses in humans and are responsible for considerable morbidity and mortality. The similarities in clinical symptoms provoked by these pathogens make diagnosis difficult. Thus, early, rapid and accurate diagnosis will be crucial in patient management and in the control of these diseases. In this study, a microfluidic lab-on-chip integrating multiplex molecular amplification and DNA microarray hybridization was developed for simultaneous detection and species differentiation of 26 globally important tropical pathogens. The analytical performance of the lab-on-chip for each pathogen ranged from 10^2^ to 10^3^ DNA or RNA copies. Assay performance was further verified with human whole blood spiked with *Plasmodium falciparum* and Chikungunya virus that yielded a range of detection from 200 to 4×10^5^ parasites, and from 250 to 4×10^7^ PFU respectively. This lab-on-chip was subsequently assessed and evaluated using 170 retrospective patient specimens in Singapore and Thailand. The lab-on-chip had a detection sensitivity of 83.1% and a specificity of 100% for *P. falciparum*; a sensitivity of 91.3% and a specificity of 99.3% for *P. vivax*; a positive 90.0% agreement and a specificity of 100% for Chikungunya virus; and a positive 85.0% agreement and a specificity of 100% for Dengue virus serotype 3 with reference methods conducted on the samples. Results suggested the practicality of an amplification microarray-based approach in a field setting for high-throughput detection and identification of tropical pathogens.

## Author Summary

Tropical diseases consist of a group of debilitating and fatal infections that occur primarily in rural and urban settings of tropical and subtropical countries. While the primary indices of an infection are mostly the presentation of clinical signs and symptoms, outcomes due to an infection with tropical pathogens are often unspecific. Accurate diagnosis is crucial for timely intervention, appropriate and adequate treatments, and patient management to prevent development of sequelae and transmission. Although, multiplex assays are available for the simultaneous detection of tropical pathogens, they are generally of low throughput. Performing parallel assays to cover the detection for a comprehensive scope of tropical infections that include protozoan, bacterial and viral infections is undoubtedly labor-intensive and time consuming. We present an integrated lab-on-chip using microfluidics technology coupled with reverse transcription (RT), PCR amplification, and microarray hybridization for the simultaneous identification and differentiation of 26 tropical pathogens that cause 14 globally important tropical diseases. Such diagnostics capacity would facilitate evidence-based management of patients, improve the specificity of treatment and, in some cases, even allow contact tracing and other disease-control measures.

## Introduction

Many infectious diseases are more prevalent in the tropical and subtropical regions where ecological, geographical and socioeconomic factors facilitate their propagation. The high diversity of such tropical pathogens include bacteria, fungi, helminths, parasites, and viruses that mirrors the rich biodiversity in the tropics and sub-tropical regions [1]–[3]. Many of these pathogens are transmissible through an insect vector or an invertebrate host [4]–[7], and transmission is affected by climate that can significantly influence vector behavior and physiology [8], including the extrinsic incubation period of vector-borne pathogens [9], [10]. Furthermore, global changes such as anthropogenic climate change and climate variability, habitat encroachment by the growing human population, volume of international travel, migration, trade and pollution create new opportunities for microbial spread [11]–[13].

The world is subjected to a plethora of tropical pathogens. Table 1 provides an overview of 14 tropical diseases, stratified into protozoan, bacterial, and viral infections that are globally important. However, some of these tropical diseases are often intimately connected to paucity of local and global burden estimates, poverty, geographical isolation and lack of coordinated approaches for disease controls [14]. Firstly, there are protozoan infections: malaria, which remains one of the most devastating and difficult parasitic diseases to be controlled and further threatened by the emergence and spread of resistance to anti-malarial drugs [15]–[17]; Chagas disease which is one of the most neglected tropical disease with a lifelong infection [18]–[20]; and human African trypanosomiasis with 60 million people at risk in Africa [21]–[23]. Next are bacterial infections: leptospirosis, which has been identified as one of the most widespread zoonosis in the world, exemplified by outbreaks in rural and urban environments [24]–[27], and more recently, emerged as a disease of the adventure traveler [28]; meliodosis that has been reported with a global distribution [29], [30]; and salmonellosis, which causes enteric fever and has a high global incidence [31]. Finally, the most prevalent infections are those of viral origins: Chikungunya fever in the Indian Ocean islands, the Indian subcontinent, southeast Asia, Africa, Europe and its emergence in the Americas [32]–[37]; Dengue fever including the emergence of dengue hemorrhagic fever [38]–[43]; West Nile fever in America [44], [45] and the increasing extensive distribution through Africa, Middle East, Europe and Asia [46]; Japanese encephalitis in Australasia [47] and in Asia [48]; yellow fever in West and Central Africa [49]; high incidence rates of hand, food and mouth disease in Asia [50]–[53]; Rift valley fever which has spread to Yemen, Saudi Arabia, northern Egypt and the French island of Mayotte [54]; and Hantavirus hemorrhagic fever which can cause serious diseases in humans with mortality rates of 12% (hemorrhagic fever with renal syndrome) and 60% (Hantavirus pulmonary syndrome) in some outbreaks [4], [55]. Despite being medically important, the incidence rates of some of these diseases are grossly underestimated and this reflects the clinical index of suspicion of the diseases which could have resulted from a lack of access to rapid diagnostics [18], [25], [29].

**Table 1:** Tropical diseases, stratified by protozoal, bacterial, and viral infections, including causative agents, and current endemic areas.

Tropical disease	Causative agent	Endemic areas
**Protozoan infections**		
Human African trypanosomiasis	*Trypanosoma brucei gambiense*, *T. brucei rhodesiense* (*T. brucei*)	Africa
Chagas disease	*Trypanosoma cruzi* (*T.cruzi*)	Latin America
Malaria	*Plasmodium falciparum* (*P. falciparum*), *P. knowlesi*, *P. malariae*, *P. ovale*, *P. vivax*)	Global distribution
**Bacterial interactions**		
Leptospirosis	Pathogenic *Leptospira* group	Global distribution
Melliodosis	*Burkholderia pseudomallei*	Global distribution
Salmonellosis	Enteric fever: *S. enterica* serovars *S. Typhi*, *S. Paratyphi* (*S. enterica*)	Global distribution
**Viral infections**		
Chikungunya fever	*Chikungunya virus* (CHIKV)	Reunion island, South-East Asia
Dengue fever	*Dengue virus* (DENV) (serotype 1 to 4)	Global distribution
Japanese Encephalitis	*Japanese Encephalitis virus* (JEV)	South-East Asia, Indian subcontinent, sporadically in Northern Australia
West Nile fever	*West Nile virus* (WNV)	
Yellow Fever	*Yellow Fever virus* (YFV)	West and Central Africa, America
Hand, foot and mouth disease	Human *enterovirus A EV-71* (EV71)	Global distribution
Viral haemorrhagic fevers	*Bunyaviridae*: *Hantaviruses* including *Dobrava-Belgrade virus* (DOBV), *Hantaan virus* (HTNV), *Seoul virus* (SEOV), *Puumala virus* (PUUV), *Tula virus* (TULV), *Andes virus* (ANDV)	Global distribution
Rift Valley fever	*Rift Valley virus* (RVV)	Arabian Peninsula and Africa

The global spread of tropical diseases emphasizes the importance of preparedness to address them. The first goal of this preparedness is fast and accurate diagnosis of medically important diseases. Differential diagnosis is based mainly on clinical examination, taking into account which diseases are locally prevalent, potential exposure, and the relevant travel history. However, the similarity and the non-specific nature of the symptoms provoked by many tropical pathogens (Table 1) complicates correct diagnosis by classical clinical observations [25], [56]–[61]. Yet, a correct diagnosis is necessary to institute effective control measures, from timely therapeutic intervention [62], [63], to effective treatment [64] and effective clinical management in deploying appropriate community-wide control measures to improve the patients' clinical outcome, disease mapping, impact monitoring, and post-elimination surveillance. Correct diagnosis can only be determined through reliable laboratory-confirmed detection and identification of tropical pathogens in clinical specimens.

Polymerase chain reaction (PCR) has been used in the diagnosis of several infectious diseases [51], [65]–[70] as it is a highly specific and sensitive method for molecular detection [71]–[73]. Moreover, much progress has been made with molecular multiplexing [74]–[78]. With the advent of microarray technology which permits simultaneous detection of a given sequence in a sample by hybridization to thousands of defined probes [79], amplification and microarray integrated assays have been made possible [74], [80]–[82].

In this study, microfluidic technology was combined with reverse transcription (RT), PCR amplification, and microarray hybridization to develop a silicon based micro electro mechanical systems (MEMS) integrated lab-on-chip that can simultaneously detect and differentiate between 26 pathogen species (including bacteria, parasites and viruses) that cause 14 tropical diseases. The detection platform is composed of the disposable lab-on-chip, a temperature control system (TCS) for the accurate control of thermal process and an optical reader for the fluorescence microarray image acquisition. The ability of the lab-on-chip to provide a “blood-to-diagnosis” solution in the detection of known and divergent pathogens was demonstrated on retrospective patient specimens. This system allows the simultaneous identification and discrimination of a large number of candidate tropical pathogens. It is undoubtedly a potential game-changer in the field of molecular diagnostics, as it provides an effective and rapid means to establish the presence of defined potential pathogens.

## Materials and Methods

### Ethics statement

The use of human samples was approved by the National Healthcare Group's Domain-Specific Ethics Review Board (DSRB reference no. B/08/026), and written informed consent was obtained from all participants. Approval was also obtained for the use of archived samples from The Oxford Tropical Research Ethics Committee (OxTREC) as part of the surveillance routine.

### Patient cohort

Plasma samples from 30 PCR-confirmed Chikungunya virus (CHIKV) patients who were admitted to the Communicable Disease Centre at Tan Tock Seng Hospital (TTSH) during the outbreak from August 1 to September 23, 2008 [83], [84] were included. Plasma samples were also collated from 10 healthy donors with informed consent (DSRB reference no. B/08/026) and used as negative controls. RNA samples were extracted using the QIAamp viral RNA mini kit (Qiagen, Hilden, Germany), according to manufacturer's instructions.

One hundred and twenty five archived nuclei acid samples extracted from specimens at the Shoklo Malaria Research Unit (SMRU) clinic on the Thai-Burmese border between 1999 and 2011 as part of two surveillance studies [16], [85] were included. DNA extracts from packed red blood cells obtained from patients (refugees and migrants) with a clear malaria diagnosis (part of the malaria burden observational study) were tested with the lab-on-chip assay. The sensitivity and specificity of the chip assay was then determined against microscopy diagnosis used by the Thailand clinics [16]. Non-malaria specimens collected from patients presenting with undifferentiated febrile illness were also evaluated with the lab-on-chip. Viral RNA extracted from acute plasma specimens that had been stored at −80°C since 2008 were used. These had previously been tested with a range of tests including Dengue RT-PCR [85]. In addition, whole blood samples from 5 native healthy volunteers were extracted using the DNeasy blood and tissue kit and QIAamp viral RNA mini kit (Qiagen) and used as negative controls.

### Human parasites

Cultures of the 3D7 clone of the NF54 strain of *Plasmodium falciparum* (*P. falciparum*) were performed using sealable flasks with RPMI-HEPES medium at pH 7.4, supplemented with 50 mg/mL hypoxanthine, 25 mM NaHCO_3_, 2.5 mg/mL gentamicin, and 0.5% (weight/volume) Albumax II (Gibco, Singapore) in an atmosphere containing 5% CO_2_, as previously described [86], [87].

### Virus isolate

The CHIKV isolate used in this study was originally isolated from a French patient returning from Reunion Island during the 2006 CHIKF outbreak [88]. After passages in Vero-E6 cultures, virus stocks were washed, and precleared by centrifugation before storing at −80°C. All virus stocks were titered by plaque assay and quantified by quantitative RT-PCR (qRT-PCR) as previously described [89], [90].

### Design of primers and probes

Target gene sequences of each pathogen (Table S1 in Text S1) were first obtained from Genbank database. Sequence alignments were performed using the ClustalW algorithm [91] in MegAlign (DNAStar, Inc., Madison, WI). A consensus sequence representing clinically relevant strains (Table S1 in Text S1) was created for each pathogen. Each target oligonucleotide sequence was designed through multiple, successive steps of evaluation of candidate sequences, based on user-defined criteria, followed by analysis with Basic Local Alignment Tool (BLAST) [92] against the nucleotide sequence database (nr/nt) [93] for non-target genomes potentially present in the specimen that could cause interference. Genus-specific PCR primers were designed for all chosen target genes sequences as previously described [94], [95]. Genus-specific (for *Plasmodium*, Flaviviruses and Hantaviruses) and species-specific capture probes were selected to target 2 to 4 regions of the targeted gene to confirm specificity and to overcome the problem of poor hybridization within the amplicon as a result of strain-specific gene polymorphisms. Efforts to improve specificity included the design of short length capture probes of 20 to 30 nucleotides in line with other studies which have shown that shorter length probes showed higher specificity [96].

### Generation of quantitative molecular standards

For each pathogen, a PCR product encompassing the targeted region was prepared using the consensus sequence and cloned into the T7 polymerase expression vector pGEMT-easy (Promega, Madison, WI) as described [70]. Serial diluted plasmid DNA or *in vitro*-transcribed RNA from respective quantified stocks was used as the DNA copy number control for DNA pathogens or RNA copy number control for RNA pathogens.

### Lab-on-chip assay

The lab-on-chip was manufactured on a silicon wafer based on MEMS and mounted on a printed circuit board (PCB) support that provides mechanical, thermal, and electrical connection [94], [95], [97] (Figure S1 in Text S1). It encompassed two silicon microreactors (12 µL) connected to a microarray chamber. The microarray chamber (3.5 mm×9.0 mm) contains 126 spots consisting of duplicate oligo-probes spotted onto the surface through a piezo-array system [95] to ensure that differential signals do not occur by chance. The enzymatic thermal cycling and hybridization reactions on the lab-on-chip are performed by the electronic TCS. Tropical pathogen detection was split into two chip versions to be subjected to two different multiplex reactions; DNA chip with a customized microarray layout specific for DNA pathogens and RNA chip specialized for RNA pathogen detection.

PCR was performed on a DNA chip in a constituted reaction of 200 nM forward and 500 nM Cy5-conjugated reverse primers in 23 µL final volume using the QuantiTect multiplex RT-PCR NoROX kit (Qiagen). Amplification was carried out with initial denaturation at 90°C for 15 min, followed by 45 cycles of 95°C for 15 sec, 60°C for 40 sec, and 72°C for 30 sec, then final extension at 72°C for 60 sec.

RT-PCR was carried out on the RNA chip using SuperScript III one-step RT-PCR system with platinum Taq (Life Technologies) in a 23 µL reaction volume containing a concentration of forward and Cy5-conjugated reverse primers in the range of 200 nM to 700 nM. After a 20-min reverse transcription step at 50°C, enzyme activation was initiated at 95°C for 120 sec, followed by denaturation at 95°C for 10 sec. Amplification was performed in a manner of touch down PCR to enhance the specificity of the initial primer-template duplex formation and hence specificity of the final PCR product [98]. The annealing temperature in the initial cycle was initiated at 60°C (5°C above the average melting temperature of the primers for RNA pathogen detection). In the subsequent 10 cycles, the annealing temperature was decreased in steps of 1°C/cycle until a temperature was reached to 50°C, and followed by extension at 72°C for 50 sec. Following these 10 cycles, 40 cycles with a temperature of 95°C for 15 sec, annealing temperature of 56°C for 40 sec, and then a final extension for 50 sec at 72°C completed the program.

Upon completion of PCR or RT-PCR, denaturation of amplicons proceeded at 95°C for 3 min, followed by hybridization at 58°C for 30 min. The lab-on-chip was washed and spin dried. The dried chip was scanned in the optical reader [95] (Veredus Laboratories) which has an excitation filter for Cy5. Accompanied software analysis was based on hybridization of amplicons to target-specific capture probes with the highest signals expected from a perfect match. Spot segmentation and intensity calculation of the microarray image was performed by overlaying a virtual grid over the microarray image using the corner features as reference points.

### Microarray interpretation criteria

For positive detection of *Plasmodium* parasites, Flaviviruses and Hantavirus, at least 1 out of 2 genus-specific probes must give a positive signal to indicate the presence of the respective genera, and at least 50% of species-specific probes must hybridize for species differentiation (Table S1 in Text S1). For the rest of the pathogens, at least 2 out of 3 pathogen-specific probes must give a positive signal for a positive detection of the pathogen (Table S1 in Text S1).

### Analytical performance and limit of detection (LoD) tests

The detection threshold and specificity of the lab-on-chip assay was evaluated by using 4 µL of quantitative standards (to cover a range of 10^1^ to 10^4^ copies per chip for each pathogen) and assessing the signal intensity and presence of cross hybridization at each copy number. Triplicates were run to ensure intra-experimental reproducibility. The lowest titer (DNA or RNA copies per chip) with 2 or more out of 3 chips positive for the assayed pathogen was further expanded to another 21 replicate runs to confirm the LoD which was the indicated titer that would yield more than 95% positive detection, as well as to evaluate inter-assay reproducibility.

### Spiked samples for extraction and assay performance

Sorted *P. falciparum* parasites were serial diluted in phosphate-buffered saline (PBS) and added to whole blood to obtain spiked samples with final concentrations of 1 to 10^3^ parasites/µL [87]. In parallel, CHIKV virus stock was serial diluted before spiking into aliquots of whole blood to cover 1 to 10^5^ PFU/µL. Spiked experiments were repeated twice for inter-experimental reproducibility. Sensitivity of the chip assay was compared with that of nested PCR [99] or qRT-PCR [70] respectively. The volume of the isolated nuclei acid subsequently used in for all comparison assays was kept constant at 4 µL.

### Statistics

All statistical analyses were performed using Prism 6.03 (GraphPad Software, Inc., La Jolla, CA). Lab-on-chip outcome on previously laboratory-confirmed samples was analyzed using Fisher exact test. P values less than 0.05 were considered statistically significant.

## Results

### Detection of tropical pathogens by lab-on-chip

**Figure 1:**
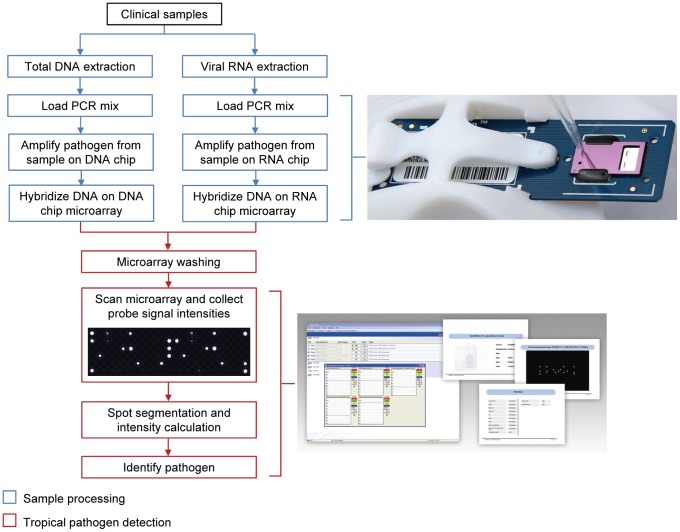
Schematic illustration of tropical pathogen detection workflow. Sample processing steps included the isolation of total DNA and viral RNA from clinical samples, followed by amplification of extracted nuclei acids on the lab-on-chip, and hybridization of amplicon to target-specific capture probes (represented in blue). Both the amplification and hybridization processes are performed on the lab-on-chip. The steps leading to tropical pathogen identification (as represented in red) comprised of washing and drying of the chip, subsequent reading of the microarray in the optical reader, and software analysis of the microarray image.

The objective of developing a portable microfluidic integrated lab-on-chip (Figure S1 in Text S1) was to provide a seamless one-time screening test for multiple tropical pathogens that exhibit similar or non-specific symptoms. Twenty-six pathogen species that cause 14 globally important but yet neglected tropical diseases (Table 1 and Table S1 in Text S1) were considered for the panel. A typical workflow for the detection of these pathogens was defined. It comprises of a processing step (blue) that includes the sample extraction and reaction setup. This is then followed by the on-chip identification and differentiation (red) (Figure 1) to ensure accurate implementation of the assay. Microarray spots were simultaneously assessed to calculate differences in signal intensities, thereby identifying unique patterns (Figure S2 in Text S1). Hybridization to a series of target-specific probe sets provided presence/absence information for the tropical pathogen, while also revealing the species of the causative agent (Figures S2, S3 in Text S1).

### Analytical sensitivity and specificity of the tropical pathogens lab-on-chip

**Figure 2:**
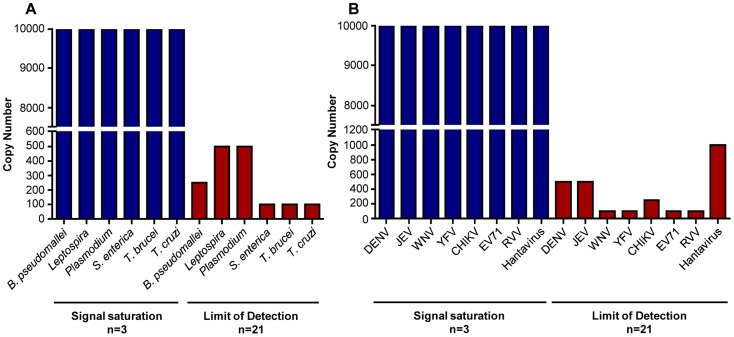
Limit of detection (LoD) of DNA and RNA pathogens on lab-on-chip assay. (A) DNA lab-on-chip has a minimum detection threshold from 10^2^ to 5×10^2^ DNA copies per reaction, while that of (B) RNA lab-on-chip is 10^2^ to 10^3^ RNA copies per reaction. The data was obtained from chips performed independently with 10^4^ copies of respective DNA or RNA quantitative standards per reaction (blue bar, n = 3) for which signal saturation for target-specific capture probes' hybridization was observed, and at LoD (red bar, n = 21).

The rationale of the analytical evaluation of the lab-on-chip was to define the LoD of the assay for all the pathogens. LoD of the lab-on-chip was determined as the lowest copy number which, in terms of plasmid copy for DNA or RNA transcript copy for RNA, when added to the chip, led to more than 95% positive pathogen identification outcome. Table S1 in Text S1 shows the lowest detectable dilution for each pathogen. The results revealed an individual sensitivity that ranged from 10^2^ to 10^3^ copies per chip (Figure 2). Target-specific hybridization signal saturation was observed at concentrations as low as 10^4^ copies for all the pathogens (Figure 2). Notably, a highly sensitive detection range of 3 orders of magnitude between LoD and signal saturation was achieved for most of the tropical pathogens, mainly *S. enterica*, *T. brucei* and *T. cruzi* under the DNA pathogen category, together with RNA viruses such as West Nile virus, yellow fever virus, Enterovirus 71 and rift valley virus (Figure 2). Although a narrow detection range of 10 copies was observed for Hantaviruses with LoD at 10^3^ copies, the rest of the pathogens stayed within the broad detection range of approximately 2 orders of magnitude. It should be noted that to date, cases of Hantavirus infections in patients yielded very low or non-detectable viral load levels [55], [100]. Probe specificity evaluation showed no significant cross reactivity (Figures S2 and S3 in Text S1).

### Assay sensitivity was further investigated with nucleic acid extraction efficiency

The efficiency of a detection assay is often dependent on the efficiency of the nuclei acid extraction method from clinical specimens [101], [102]. Some methods may even interfere with the PCR reaction [103], [104]. The purpose of the investigation was to assess the efficiency of the extraction method and the sensitivity of the lab-on-chip using *P. falciparum* and CHIKV as targets.

**Figure 3:**
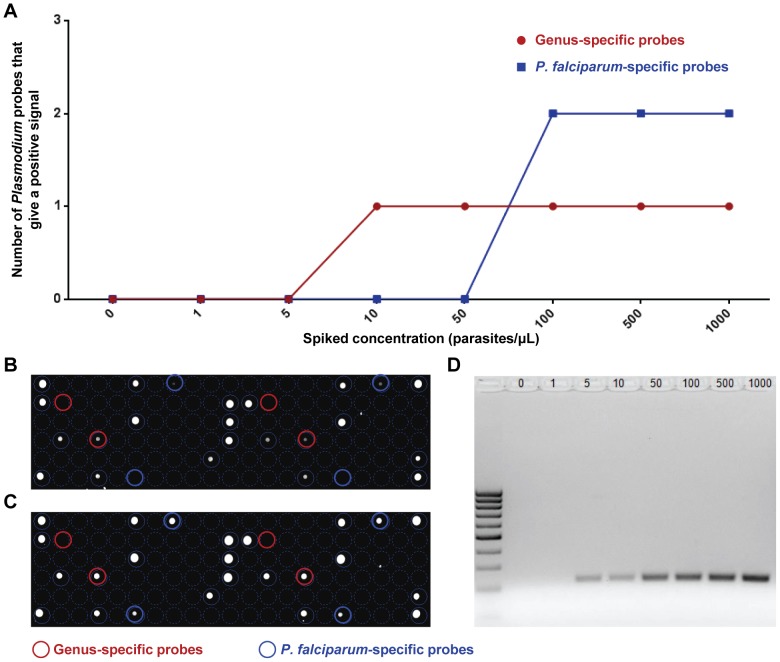
DNA extraction and amplification strategy in the detection of *P. falciparum*. DNA extracted (4 µL) from respective spike tests of 1 to 10^3^ parasites/µL was subjected to DNA lab-on-chip amplification or nested PCR assay. (A) Summary of *Plasmodium* genus-specific and *P. falciparum* specific positive probes for respective spiked concentrations (n = 2). The x-axis showed the different spiked concentrations used. The y-axis represented the number of positive probes at each dilution tier. Genus-specific probes are represented in red, while species-specific probes are in blue. Hybridization profiles of DNA lab-on-chip of extracted DNA from (B) 50 parasites/µL and (C) 100 parasites/µL spiked samples are shown. *Plasmodium* genus-specific probes are marked in red, while *P. falciparum* specific probes are marked in blue. Nested PCR can detect *P. falciparum* in spiked samples as low as 5 parasites/µl. The PCR products from the nested PCR assay were run on a 2% agarose gel electrophoresis. (D) Lane 1 = 0, lane 2 = 1, lane 3 = 5, lane 4 = 10, lane 5 = 50, lane 6 = 100, lane 7 = 500 and lane 8 = 1000 parasites/µL spiked samples. L: PCR Sizer 100 base pair DNA Ladder.

The read-out for the lab-on-chip and that of nested PCR is illustrated in Table 2 and in Figure 3. The presence of *P. falciparum* in the extracted spiked samples was demonstrated by the presence of hybridized genus-specific and species-specific probes on the microarray for lab-on-chip, while that by nested PCR relied on the presence a PCR band on agarose gel [99]. Positive detection of *P. falciparum* by the lab-on-chip was observed at 100 parasites, while positive bands were detected at 5 parasites by nested PCR (Table 2 and Figure 3). Although the nested PCR method [99] is more sensitive with a difference of more than one log when compared to the lab-on-chip (Table 2 and Figure 3), it is more labor intensive.

**Table 2:** DNA lab-on-chip analytical sensitivity using *Plasmodium* spiked samples.

	Nested PCR	Lab-on-chip		
Spiked concentrations	Band observed on 2% agarose gel	*Plasmodium* genus probes^a^	*P. falciparum* specific probes^b^	Analysis^c^^,^ d
0 parasite/µL	ND	0	0	ND
1 parasites/µL	ND	0	0	ND
5 parasites/µL	Yes	0	0	ND
10 parasites/µL	Yes	1	0	ND
50 parasites/µL	Yes	1	0	Positive for *Plasmodium*
100 parasites/µL	Yes	1	2	Positive for *P. falciparum*
500 parasites/µL	Yes	1	2	Positive for *P. falciparum*
1,000 parasites/µL	Yes	2	2	Positive for *P. falciparum*

a shows the number *Plasmodium* genus probes (out of two) with a fluorescence signal.

b shows the number *P. falciparum* specific probes (out of two) with a fluorescence signal.

c A positive detection for *P. falciparum* would require the presence of at least one of two probes (for both genus and *P. falciparum* specific) to give a positive fluorescence signal.

d ND Not detected.

**Figure 4:**
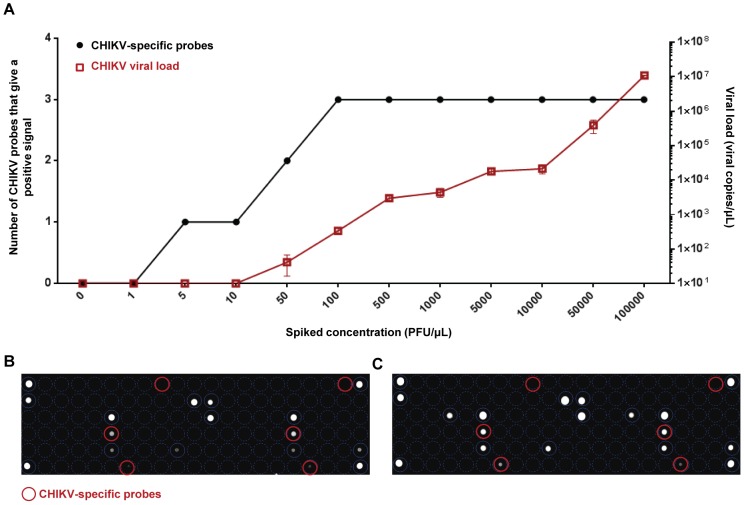
Viral RNA extraction and amplification strategy in the detection of CHIKV. RNA extracted (4 µL) from respective spikes of 1 to 10^5^ PFU/µL was subjected to RNA lab-on-chip amplification or qRT-PCR assay. (A) Summary profiles of CHIKV specific probes and viral load quantification for respective spiked concentrations (n = 2). Hybridization profiles of RNA lab-on-chip of extracted viral RNA from spiked samples of (B) 10 PFU/µL and (C) 50 PFU/µL are shown. CHIKV specific probes are marked in blue.

The estimated PFU isolated from CHIKV-spiked samples (in red) compared to the viral load derived from qRT-PCR is shown in Table 3 and in Figure 4. The detection threshold for CHIKV was 50 PFU (Figure 4B, 4C). More importantly, the sensitivity of the detection range of the lab-on-chip and viral load quantification by qRT-PCR are similar, clearly demonstrating the superiority of the lab-on-chip (Figure 4).

**Table 3:** RNA lab-on-chip analytical sensitivity using CHIKV spiked samples.

	Taqman assay	Lab-on-chip	
Spiked concentrations	Viral load quantification after extraction (viral copies)	CHIKV specific probes^a^	Analysis^b^^,^ c
0 PFU/µL	0	0	ND
1 PFU/µL	0	0	ND
5 PFU/µL	0	1	ND
10 PFU/µL	0	1	ND
50 PFU/µL	59	2	Positive for CHIKV
100 PFU/µL	292	3	Positive for CHIKV
500 PFU/µL	3,311	3	Positive for CHIKV
1,000 PFU/µL	3,515	3	Positive for CHIKV
5,000 PFU/µL	20,306	3	Positive for CHIKV
10,000 PFU/µL	25,616	3	Positive for CHIKV
50,000 PFU/µL	50,149	3	Positive for CHIKV
100,000 PFU/µL	1,092,983	3	Positive for CHIKV

a shows the number CHIKV specific probes (out of two) with a fluorescence signal.

b A positive detection for CHIKV would require the presence of at least one of the two CHIKV specific probes to give a positive fluorescence signal.

c ND Not detected.

### Clinical validation of the tropical pathogens lab-on-chip in field settings

**Figure 5:**
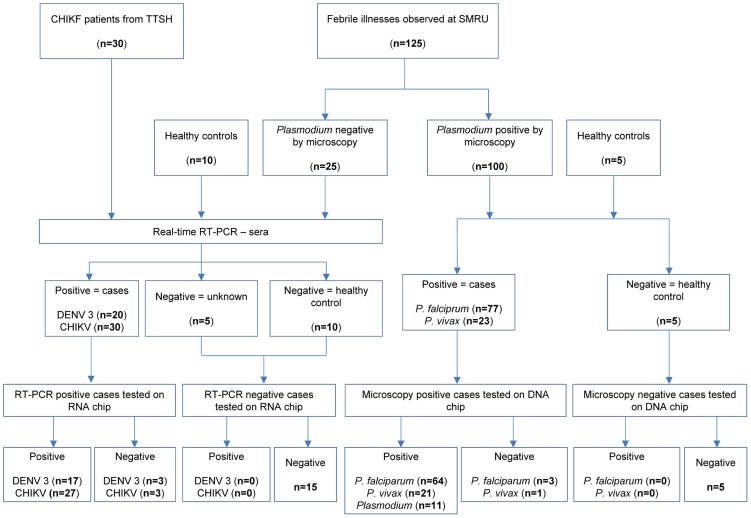
Flowchart detailing the screening and order of diagnostic testing of 160 samples received in Singapore and Thailand. Specimens positive for Plasmodium parasites were tested with lab-on-chip to evaluate the performance of the assay. Non-malaria samples were evaluated for CHIKV and DENV and subsequently tested with lab-on-chip assay for diagnostic methodology evaluation.

**Figure 6:**
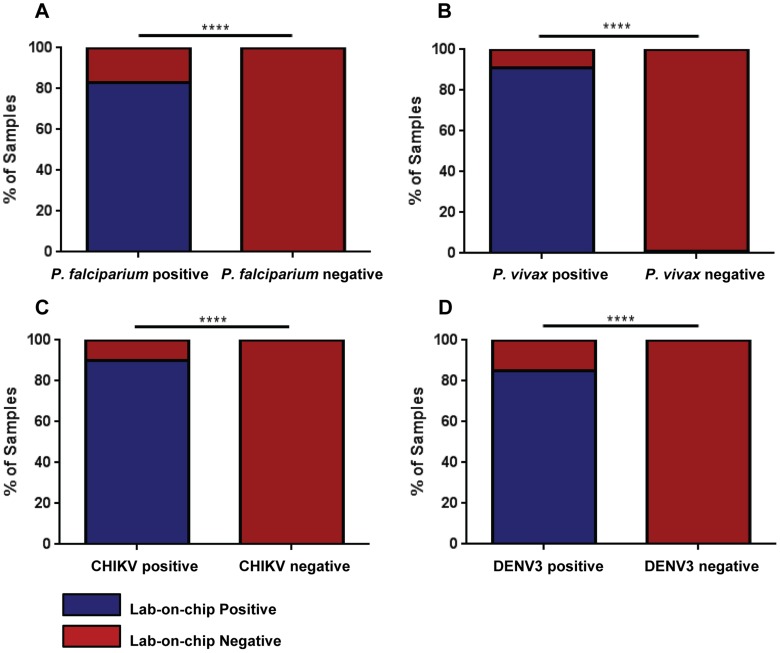
Association of microscopy and RT-PCR detection with lab-on-chip outcome. The outcome of the tropical pathogen chip test on clinical samples previously confirmed by microscopy or RT-PCR. (A) *P. falciparum*. (B). *P. vivax*. (C) CHIKV. (D). DENV 3. Histograms show the percentage of samples tested positive for *P. falciparum* (n = 64), *P. vivax* (n = 21), CHIKV (n = 27), and DENV 3 (n = 17) by DNA or RNA lab-on-chip. Statistical significance was measured using 2-sided Fisher exact test between the number of samples tested positive or negative for the respective pathogens by the chip on previously laboratory-confirmed samples (by reference methods). ****P<.0001.

In order to assess the clinical performance of the assay, the lab-on-chip was evaluated on retrospective clinical specimens to compare its diagnostic capability with reference methods. The screening and order of diagnostic testing of 170 samples received in Singapore and Thailand are illustrated in Figure 5. Sixty-four out of 77 *P. falciparum* positive samples and 21 out of 23 *P. vivax* positive samples were concordant with the microscopic diagnosis (Tables 4, 5). The sensitivity and the specificity for the detection of *P. falciparum* was 83.1% (72.9% to 90.7%) and 100% (96.1% to 100%) (Table 4, Figure 6), and that of *P. vivax* was 91.3% (71.9% to 98.9%) and 99.3% (96.3% to 99.9%) (Table 5, Figure 6). Fourteen *P. falciparum* positive samples with low levels of parasitemia did not yield a positive detection for *P. falciparum*, but 11 out of the 14 were tested positive for *Plasmodium*. Although species differentiation was not achieved with these 11 samples, the assay did provide a diagnosis for *Plasmodium*.

**Table 4:** Clinical performance of DNA chip on *P. falciparum*.

Data analyzed	Microscopy positive	Microscopy negative	DNA chip total
DNA chip positive	64	0	64
DNA chip negative	13	93	106
Microscopy total	77	93	170
Clinical sensitivity	83.1% (72.9% to 90.7%)		
Clinical specificity	100% (96.1% to 100%)		
Positive predictive value	100%		
Negative predictive value	87.7%		

DNA lab-on-chip results for 77 *P. falciparum* clinical isolates out of 170 specimens were compared with results from microscopy.

**Table 5:** Clinical performance of DNA chip on *P. vivax*.

Data analyzed	Microscopy positive	Microscopy negative	DNA chip total
DNA chip Positive	21	1	22
DNA chip Negative	2	146	148
Microscopy total	23	147	170
Clinical sensitivity	91.3% (71.9% to 98.9%)		
Clinical specificity	99.3% (96.3% to 99.9%)		
Positive predictive value	95.5%		
Negative predictive value	98.6%		

DNA lab-on-chip results for 23 *P. vivax* clinical isolates out of 170 specimens were compared with results from microscopy.

The validation also yielded a good positive 90.0% agreement (73.5% to 97.9%) and excellent specificity 100% (97.4% to 100%) for the CHIKV detection (Table 6, Figure 6). Finally, the assay showed an average positive 85% agreement (62.1% to 96.8%) (17 out of 20 DENV positive samples) and a specificity of 100% (97.5% to 100%) for DENV 3 detection (Table 7, Figure 6). The 3 CHIKV samples that were not detected positive by the lab-on-chip were that with low viral load of less than 10^2^ viral copies/µL quantified by qRT-PCR [70]. All healthy donor samples tested were negative.

**Table 6:** Clinical performance of RNA chip on CHIKV.

Data analyzed	qRT-PCR positive	qRT-PCR negative	RNA chip total
RNA chip Positive	27	0	27
RNA chip Negative	3	140	143
qRT-PCR total	30	140	170
Positive percent agreement	90.0% (73.5% to 97.9%)		
Specificity	100% (97.4% to 100%)		
Positive predictive value	100%		
Negative predictive value	97.9%		

RNA lab-on-chip results for 30 CHIKV clinical isolates out of 170 specimens were compared with results from qRT-PCR.

**Table 7:** Clinical performance of RNA chip on DENV.

Data analyzed	RT-PCR positive	RT-PCR negative	RNA chip total
RNA chip Positive	17	0	17
RNA chip Negative	3	150	153
RT-PCR total	20	150	170
Positive percent agreement	85.0% (62.1% to 96.8%)		
Specificity	100% (97.5% to 100%)		
Positive predictive value	100%		
Negative predictive value	98.0%		

RNA lab-on-chip results for 20 DENV 3 clinical isolates out of 170 specimens were compared with results from RT-PCR.

## Discussion

While every disease presents specific diagnostic challenges, clinical needs associated with specificity, sensitivity, total analysis time, and implementation would eventually impact the design and development of the diagnostic method. In this study, an integrated strategy for miniaturizing and simplifying complex laboratory assays for the detection of 14 globally important tropical diseases stood out favorably in terms of seamless implementation and pathogen coverage compared to conventional laboratory diagnostic methodologies.

The mainstay to detect protozoan infections such as Chagas disease, human African trypanosomiasis, and malaria infection relies in the conclusive visualization of the parasites in blood [18],[21],[105]. The reliable identification of these infections requires high quality training in specimen preparation and a competency in identifying the parasites when compared to the facile interpretation of the lab-on-chip microarray analysis.

Bacteria culture remains as one of the most effective procedures in identifying bacterial infections [106]–[108] and is also crucial in generating pools of clinical strains for pathogenesis studies. However, the process is labor and time intensive, spanning from a few days to several weeks when compared to the lab-on-chip assay that is completed within 4 hours. It is also dependent on stringent transport conditions and well-maintained equipments to maintain specimen viability.

While methods based on serological reactivity to pathogen-specific antibodies [109]–[111] have been developed to identify several viral infections and are useful in differentiating viruses within the same family or genus, cross reactivity remains a conflicting issue [100], [112], [113]. In spite of cross reactivity issues, serology is still widely used to confirm diagnosis due to limitations in the detection window of nucleic acids [83], [85], [100]. Here, the analytical performance of the lab-on-chip has highlighted its specificity with no cross reactivity observed between the 5 *Plasmodium* species, between DENV and the other 3 Flaviviruses, and among the 6 Hantaviruses, achieved in just one test. Future iterations of the lab-on-chip could include protein-based arrays as additional serology screens [114], [115] for some diseases that are clinically warranted as orthogonal diagnosis based on nucleic acid, protein, and other biomarkers will be where the field is heading.

Simultaneous laboratory screening of a clinical specimen from a patient with unspecific symptoms for as many tropical agents as possible would either lead to pathogen identification or narrow down the possible causes through elimination. However, combining the various assays for parallel screening of tropical diseases is not a feasible approach given the high diversity of the protocols with many limitations associated with each pathogen. Even though amplification microarray assays [80]–[82] have been developed to circumvent the need for parallel tests, detection in these assays was restricted to one virus family, despite an improvement in pathogen coverage, and thus still considered as low throughput. Moreover, simultaneous detection was achieved only after 3 separate amplification reactions for the 3 respective virus families [80]. Miniaturized total analysis systems [116] have evolved, that has led to miniaturized PCR devices being extensively studied [117]. A few reports have demonstrated rapid on-chip detection of Influenza A virus [118], [119] and human immunodeficiency virus [120], however the development of a miniaturized assay for the detection of multiple tropical diseases pathogens including the validation on patient specimens has yet to be demonstrated.

The design and process of the lab-on-chip evaluation was approached systematically. It was first evaluated using quantitative standards. The LoD of the lab-on-chip was shown to range from 10^2^ to 10^3^ copies and signal saturation for target-specific capture probes' hybridization was at 10^4^ copies. This observation was crucial as the efficiency of the chip to detect the relevant pathogen in a clinical sample load on the chip containing 10^4^ or more copies of that pathogen would be 100%. When considering the detection limit of the lab-on-chip of the pathogen in a clinical sample, the target concentration required to get the minimum amount of nuclei acids after sample extraction in the amplification reaction must be investigated. Comparison of the lab-on-chip with nested PCR using spiked *P. falciparum* samples and with qRT-PCR on spiked CHIKV samples has proven the efficiency of the extraction method and also emphasized a more superior trade-off between the assay's sensitivity and its utility in the systemic differentiation of *P. falciparum* and detection of CHIKV. The lab-on-chip assay's ability to detect CHIKV at 50 PFU/µL demonstrated high clinical relevance as it was shown that the mean CHIKV viral load in patients ranged between 126 to 241 PFU/µL [83].

One of the key objectives of the clinical validation was to investigate the lab-on-chip's performance and acceptability in field settings and the degree to which the results would determine the quality of the diagnosis for surveillance and patient management to improve health outcomes. The clinical validation of *P. vivax* offered a sensitivity that was equivalent to microscopy. Although there was a proportion of *P. falciparium* samples (14 out of 77 samples) with low parasitiamia that were not positively detected for *P. falciparum* on the lab-on-chip, the assay did manage to give a partial diagnosis (of the samples being *Plasmodium* positive) for 11 of these samples. Although the lab-on-chip did not positively differentiate samples with extremely low levels of parasitemia, the low parasite burden of these patients could represent the early stages of malaria. Taken together, the analytical performance of the lab-on-chip for *P. falciparum* and *P. vivax* in the range of 10^2^ copies, and the demonstration of its diagnostic utility using spiked samples and clinical specimens showed the applicability of the assay for *Plasmodium* detection.

The clinical performance of the lab-on-chip for DENV and CHIKV was comparable to RT-PCR. For DENV, comparisons among the diagnostic tests at SMRU have demonstrated RT-PCR to have the best operating characteristics (sensitivity 89%, specificity 96%, positive predictive value 94%, negative predictive value 92%) [85]. This suggested that the chip would be potentially sufficient to function as a single assay for confirmation of Dengue infection, since it allowed for accurate confirmation. Similarly, the assay sensitivity for CHIKV was on par with that of RT-PCR, and achieved a positive 90% agreement with patients' samples.

The cost of the assay compared to that of single assays is high. Advancements in the integration of the lab-on-chip with nuclei extraction capabilities [95] and a higher density microarray with reduced chip cost would provide a more cost-effective comprehensive coverage. While the lab-on-chip assay has showed that miniaturized multiplex PCR could reach the desired clinical sensitivity, future work should attempt to recalibrate the mix of multiplex primers and modify amplification cycling conditions for improved sensitivity. One of the key milestones for lab-on-chip systems would be the direct testing of clinical specimens obtained during the acute infection phase and provide accurate diagnosis to complement clinical assessments.

## Supporting Information

Text S1 **Supporting information.** This file contains the STARD Checklist, four supplementary figures and one supplementary table. **Figure S1. Lab-on-chip design.** (A) Photograph of lab-on-chip. Dimension of each chip is 75 mm in width, 25 mm in length and 1 mm thick. (B) The lab-on-chip detection platform which consists of the TCS and optical reader connected to a computer. **Figure S2. Microarray differentiation of DNA tropical pathogens on DNA chip.** Each panel is a representative experiment of 3 independent experiments performed and shows the hybridization profile of the amplified target gene fragment of the respective plasmid control of 10000 copy number. Probes marked in red are positive hybridization positional probes, while probes marked in green are positive hybridization probes. Additionally probes marked in light grey are PCR control probes. Finally, probes marked in yellow are specific probes for (A) *Burkholderia pseudomallei*. (B) *Leptospira*. (C) *P. falciparum*. (D) *P. knowlesi*. (E) *P. malariae*. (F) *P. ovale*. (G) *P. vivax*. (H) *S. enterica*. (I) *T. brucei*. (J) *T. cruzi*. Genus-specific probes are marked in orange. **Figure S3. Microarray differentiation of RNA tropical pathogens on RNA chip.** The respective panels show the hybridization profiles of the amplified target gene fragment of the following *in-vitro* transcript RNA of 10000 copies number. Probes marked in red are positive hybridization positional probes, while probes marked in green are positive hybridization probes. Additionally probes marked in light grey are RT-PCR control probes. Species-specific or pathogen-specific probes for the RNA pathogens are marked as follows: (A) YFV and ANDV. (B) DENV 1 and RVV. (C) DENV 2 and DOBV. (D) DENV 3 and SEOV. (E) DENV 4 and TULV. (F) JEV and EV71. (G) CHIKV and HTNV. (H) WNV and PUUV. Genus-specific probes are in light blue and orange. Species-specific probes are in purple and yellow. **Table S1. Lab-on-chip assay detection capacity.**
(ZIP)
