# Supplementary material for: An Integrated Lab-on-Chip for Rapid Identification and Simultaneous Differentiation of Tropical Pathogens
Source: PLoS Negl Trop Dis. 2014 Jul 31;8(7):e3043. doi: 10.1371/journal.pntd.0003043 (PMC4117454; doi:10.1371/journal.pntd.0003043)
Supplement: Text S1 — Supporting information. This file contains the STARD Checklist, four supplementary figures and one supplementary table. Figure S1. Lab-on-chip design. (A) Photograph of lab-on-chip. Dimension of each chip is 75 mm in width, 25 mm in length and 1 mm thick. (B) The lab-on-chip detection platform which consists of the TCS and optical reader connected to a computer. Figure S2. Microarray differentiation of DNA tropical pathogens on DNA chip. Each panel is a representative experiment of 3 independent experiments performed and shows the hybridization profile of the amplified target gene fragment of the respective plasmid control of 10000 copy number. Probes marked in red are positive hybridization positional probes, while probes marked in green are positive hybridization probes. Additionally probes marked in light grey are PCR control probes. Finally, probes marked in yellow are specific probes for (A) Burkholderia pseudomallei. (B) Leptospira. (C) P. falciparum. (D) P. knowlesi. (E) P. malariae. (F) P. ovale. (G) P. vivax. (H) S. enterica. (I) T. brucei. (J) T. cruzi. Genus-specific probes are marked in orange. Figure S3. Microarray differentiation of RNA tropical pathogens on RNA chip. The respective panels show the hybridization profiles of the amplified target gene fragment of the following in-vitro transcript RNA of 10000 copies number. Probes marked in red are positive hybridization positional probes, while probes marked in green are positive hybridization probes. Additionally probes marked in light grey are RT-PCR control probes. Species-specific or pathogen-specific probes for the RNA pathogens are marked as follows: (A) YFV and ANDV. (B) DENV 1 and RVV. (C) DENV 2 and DOBV. (D) DENV 3 and SEOV. (E) DENV 4 and TULV. (F) JEV and EV71. (G) CHIKV and HTNV. (H) WNV and PUUV. Genus-specific probes are in light blue and orange. Species-specific probes are in purple and yellow. Table S1. Lab-on-chip assay detection capacity. (ZIP) [file pntd.0003043.s001.zip › Tan et al 2014_Supporting Information File_Resubmit_21May2014/Tan et al 2014_Supporting Information Table S1_Resubmit_21May2014.pdf]

Supporting Information

Table S1. Lab-on-chip assay detection capacity.

Tropical disease	Tropical pathogen [a]	Target gene	Number of genus-specific probes for detection [b]	Number of species-specific probes for detection [c]	Amplicon size (bp) [d]	Amplification efficiency (%) [d, e]	Limit of detection [f, g, h]	Accession number [i]
Melliodosis	*Burkholderia pseudomallei*	Type III secretion system	NA	2 out of 3 *B. pseudomallei* specific	341	0.98	1.00E+02	AF074878, BX571966, CP000573, CP000125
Leptospirosis	*Leptospira borgpetersenenii*	LipL32	2 out of 3 *Leptospira* specific	NA	208	0.83	5.00E+02	AF181554, EU526390
	Leptospira interrogans							AF366366, AF245281, LIU89708, AY609321
	Leptospira kirschneri							AF121192, AY461917
	Leptospira noguchii							AF181556, AY461920
	Leptospira santorosai							AY461928, AF181555
	Leptospira weilii							AY461930, AY609331
Malaria	*Plasmodium falciparum*	18S small subunit ribosomal RNA	1 out of 2 *Plasmodium* specific	1 out of 2 *P. falciparum* specific	336	1.00	2.50E+02	M19173, M19172
	Plasmodium knowlesi			1 out of 2 *P. knowlesi* specific	331	0.92	1.00E+02	AY580317, U83876
	Plasmodium malariae			1 out of 2 *P. malariae* specific	340	0.79	5.00E+02	M54897, AF487999, AF488000
	Plasmodium ovale			2 out of 3 *P. ovale* specific	328	0.95	5.00E+02	L48987, AB182491, AB182489
	Plasmodium vivax			1 out of 2 *P. vivax* specific	327	0.89	1.00E+02	U03079, U83877
Salmonellosis	*Salmonella enterica*	Superblock 18 (named from in-house alignment)	NA	2 out of 3 *S. enterica* specific	201	0.91	1.00E+02	AE014613, AL513382, FM200053, CP000026, AE017220, CP000880, CP001138, CP001144, AM933172, AM933173, CP001120, CP001113, CP000886, CP000857, CP001127
Human African trypanosomiasis	*Trypanosoma brucei*	18S small subunit ribosomal RNA	1 out of 2 *Trypanosoma* specific	1 out of 2 *T. brucei* specific	296	0.86	1.00E+02	AJ009141, AJ009142
Chagas disease	*Trypanosoma cruzi*			1 out of 2 *T. cruzi* specific	272	0.87	1.00E+02	AF303660
Chikungunya fever	*Chikungunya virus* (CHIKV)	E1 glycoprotein	NA	2 out of 3 CHIKV specific	188	0.85	2.50E+02	FJ807886 and FJ807895, FJ807896–FJ807899, AF369024
Dengue fever	*Dengue serotype 1 virus* (DENV 1)	NS5 RNA-directed RNA polymerase	1 out of 2 Flavivirus specific	1 out of 2 DENV1 specific	437	0.85	2.50E+02	AF513110, EU081281, U88535
	Dengue serotype 2 virus (DENV 2)			1 out of 2 DENV2 specific			1.00E+02	AF489932, AY702038, EU081180
	Dengue serotype 3			1 out of 2 DENV3			5.00E+02	AY099336, AY858037,

	virus (DENV 3)			specific				EU081225
	Dengue serotype 4 virus (DENV 4)			1 out of 2 DENV4 specific			1.00E+02	AF289029, AY762085, AY947539
Japanese Encephalitis	*Japanese Encephalitis Virus* (JEV)			1 out of 2 JEV specific	440	0.87	5.00E+02	EU429297, FJ495189, GQ918133, NC_001437
West Nile fever	*West Nile Virus* (WNV)			2 out of 3 WNV specific		0.85	1.00E+02	AF196835, AF260968, AY268132, DQ116961, DQ211652, NC_001563
Yellow Fever	*Yellow Fever Virus* (YFV)			2 out of 3 YFV specific		0.99	1.00E+02	AY968064, AY968065, DQ118157, FJ654700, NC_002031, U21056
Hand, foot and mouth disease	*Human enterovirus A EV-71* (EV71)	Polyprotein	NA	2 out of 3 EV71 specific	238	0. 87	1.00E+02	AF316321, AF352027, AF119795, FJ607337, FJ607338, GQ994988, GQ994991, GQ892830, GQ994992
Viral haemorrhagic fever	*Dobrava-Belgrade virus* (DOBV)	S segment	1 out of 2 Hantavirus specific	1 out of 2 DOBV specific	443	0.86	1.00E+03	AJ410619, L41916, AY168576
	Hantaan virus (HTNV)			1 out of 2 HTNV specific	466	0.82	1.00E+03	FJ753396, M14626,
	Seoul virus (SEOV)			1 out of 2 SEOV specific	533	0.81	1.00E+03	AY273791, AF187082
	Puumala virus (PUUV)			2 out of 3 PUUV specific	582	0.74	1.00E+03	AJ238790, GU808825, X61035, Z48586
	Tula virus (TULV)			2 out of 3 TULV specific	595	0.75	1.00E+03	AF164094, Z30941, Z69991
	Andes virus (ANDV)			2 out of 3 ANDV specific	641	0.69	1.00E+03	AF004660, AF324902, AF291702
Rift Valley fever	*Rift Valley Virus* (RVV)	G2		2 out of 3 RVV specific	272	1.00	1.00E+02	AF134508
	Photinus pyralis (Luc)	Luciferase	NA	1 out of 2 Luc specific	183	ND	ND	M15077
	Vigna radiate (PSII)	Chloroplast-encoded	NA	1 out of 2 PSII specific	177	ND	ND	NC_013843

[a] Samples were generated by diluting purified nucleic acid templates in Tris-EDTA buffer to create 10^6 copies/μL stock solution. Starting from this concentration, 10-fold serial dilutions in Tris-EDTA buffer were prepared. [b] Not applicable (NA) for genus-specific. [c] Not applicable (NA) for species-specific. [d] Primer assessment on 7900HT fast real time PCR system. [e] $E = 10^{(-1/slope)} - 1$. [f] Lab-on-chip assay. [g] A total of 21 replicate runs were performed to confirm the confidence interval of more than 95% positive detection at the indicated titer. [h] Not disclosed (ND). [i] GenBank accession numbers of nucleotide sequences used to perform multiple sequence alignments.
