# Supplementary material for: An Integrated Lab-on-Chip for Rapid Identification and Simultaneous Differentiation of Tropical Pathogens
Source: PLoS Negl Trop Dis. 2014 Jul 31;8(7):e3043. doi: 10.1371/journal.pntd.0003043 (PMC4117454; doi:10.1371/journal.pntd.0003043)
Supplement: Text S1 — Supporting information. This file contains the STARD Checklist, four supplementary figures and one supplementary table. Figure S1. Lab-on-chip design. (A) Photograph of lab-on-chip. Dimension of each chip is 75 mm in width, 25 mm in length and 1 mm thick. (B) The lab-on-chip detection platform which consists of the TCS and optical reader connected to a computer. Figure S2. Microarray differentiation of DNA tropical pathogens on DNA chip. Each panel is a representative experiment of 3 independent experiments performed and shows the hybridization profile of the amplified target gene fragment of the respective plasmid control of 10000 copy number. Probes marked in red are positive hybridization positional probes, while probes marked in green are positive hybridization probes. Additionally probes marked in light grey are PCR control probes. Finally, probes marked in yellow are specific probes for (A) Burkholderia pseudomallei. (B) Leptospira. (C) P. falciparum. (D) P. knowlesi. (E) P. malariae. (F) P. ovale. (G) P. vivax. (H) S. enterica. (I) T. brucei. (J) T. cruzi. Genus-specific probes are marked in orange. Figure S3. Microarray differentiation of RNA tropical pathogens on RNA chip. The respective panels show the hybridization profiles of the amplified target gene fragment of the following in-vitro transcript RNA of 10000 copies number. Probes marked in red are positive hybridization positional probes, while probes marked in green are positive hybridization probes. Additionally probes marked in light grey are RT-PCR control probes. Species-specific or pathogen-specific probes for the RNA pathogens are marked as follows: (A) YFV and ANDV. (B) DENV 1 and RVV. (C) DENV 2 and DOBV. (D) DENV 3 and SEOV. (E) DENV 4 and TULV. (F) JEV and EV71. (G) CHIKV and HTNV. (H) WNV and PUUV. Genus-specific probes are in light blue and orange. Species-specific probes are in purple and yellow. Table S1. Lab-on-chip assay detection capacity. (ZIP) [file pntd.0003043.s001.zip › Tan et al 2014_Supporting Information File_Resubmit_21May2014/Tan et al 2014_Supporting Information_Figure S1 to S3_Resubmit_21May2014.pdf]

A

B

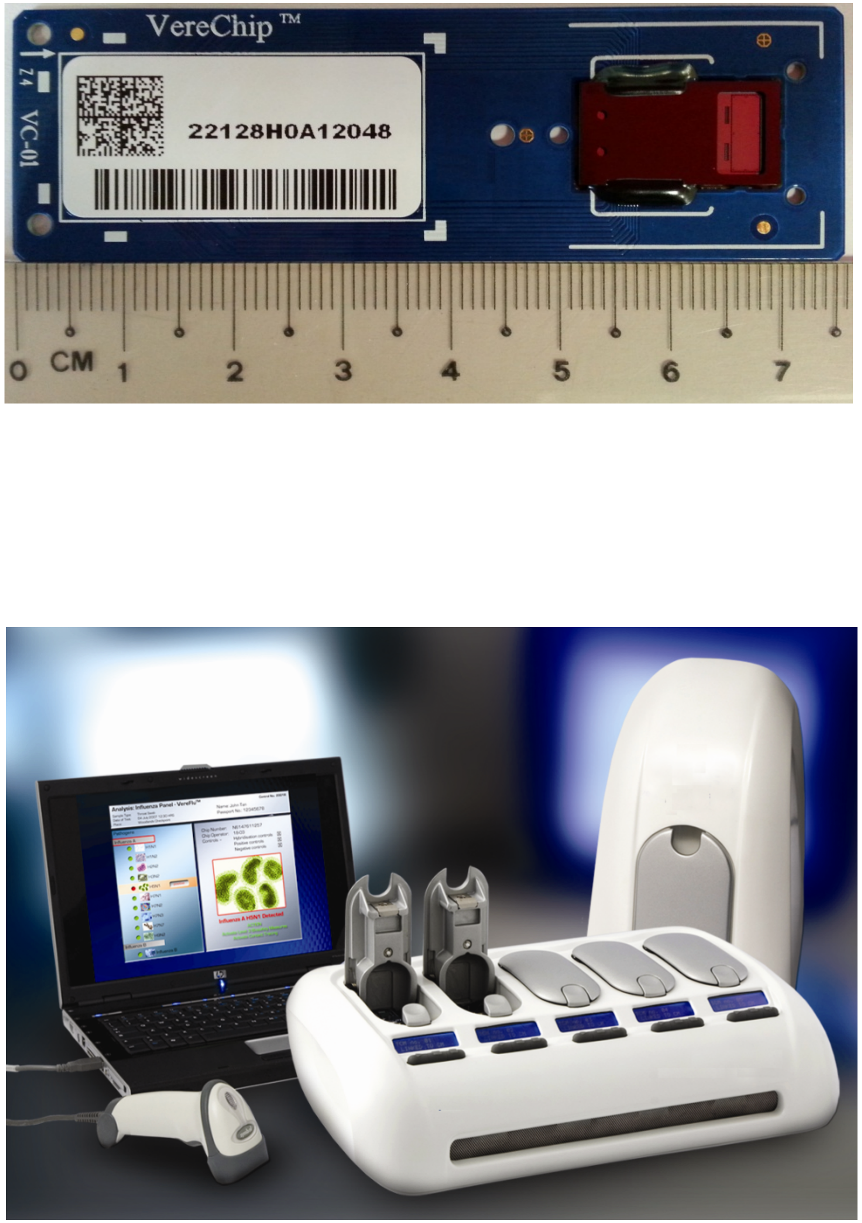

Figure S1 Tan et al., 2014

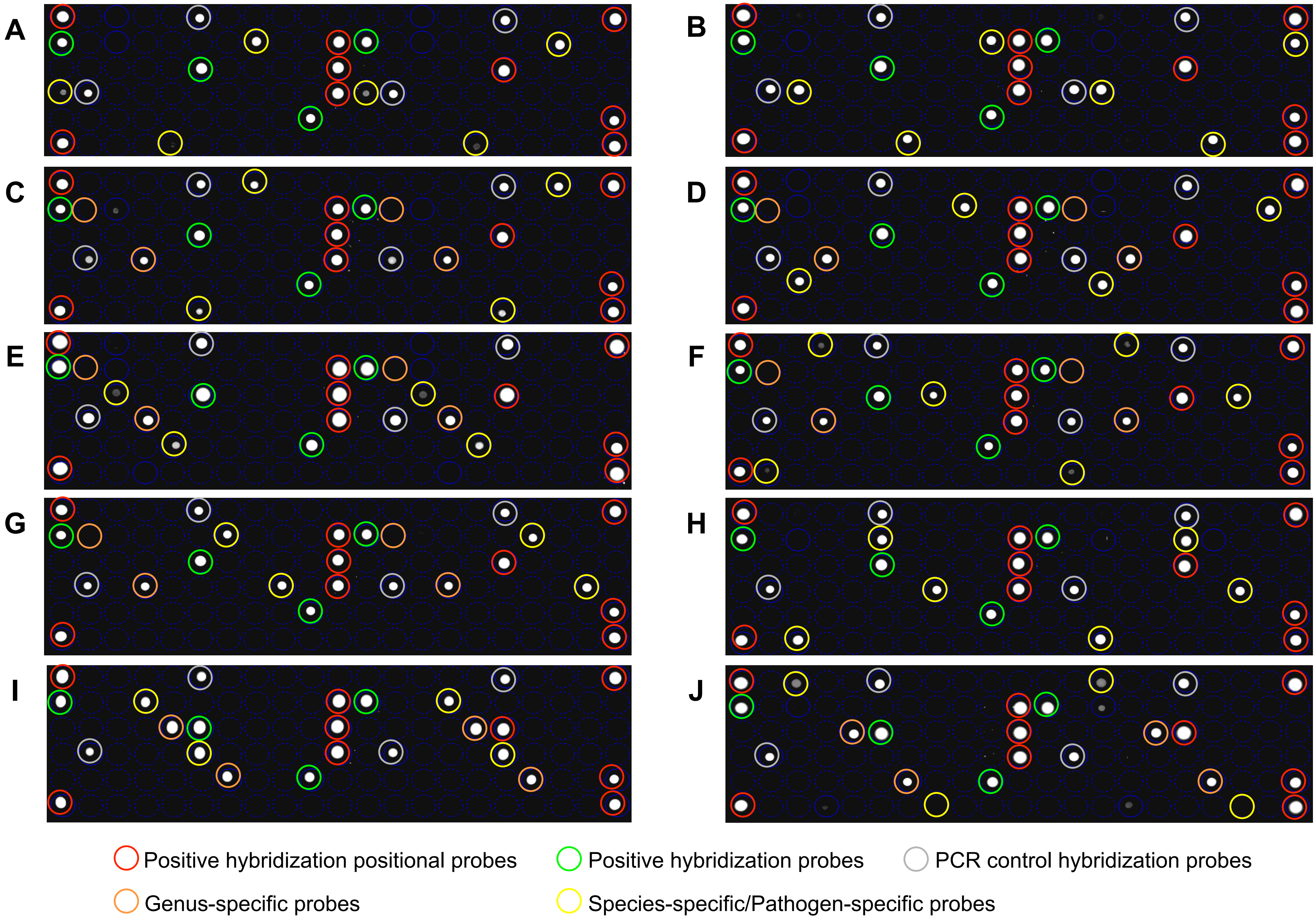

Figure S2 Tan et al., 2014

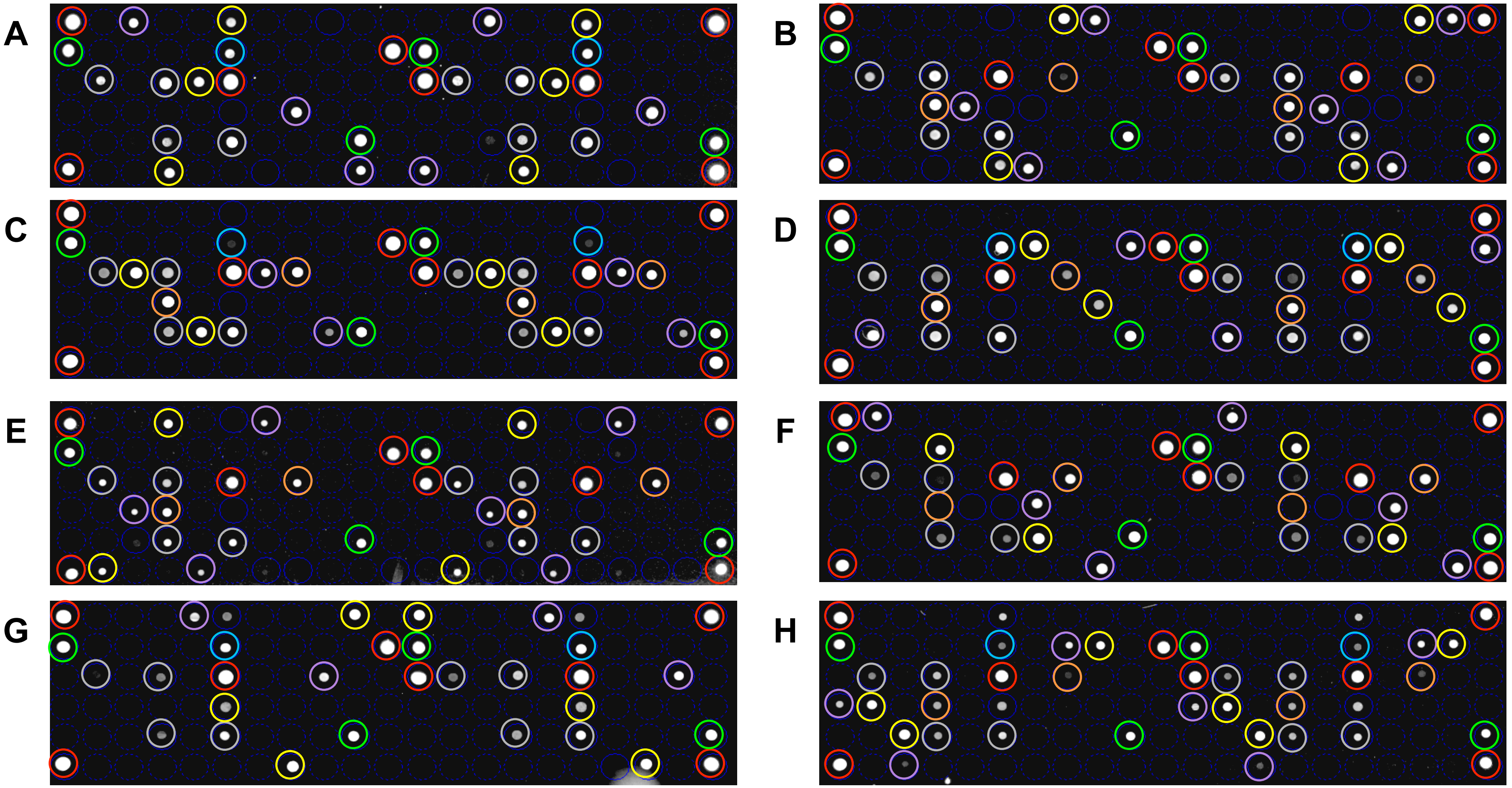

Positive hybridization positional probes

Positive hybridization probes

PCR control hybridization probes

Genus-specific probes

Species-specific/Pathogen-specific probes

Genus-specific probes

Species-specific/Pathogen-specific probes

Figure S3 Tan et al., 2014
